# Association between cigarette smoking patterns and severity of COVID-19: Findings from a study in 15 private Hospitals in Indonesia

**DOI:** 10.18332/tid/159622

**Published:** 2023-02-17

**Authors:** Emma Rachmawati, Mochamad Iqbal Nurmansyah, Izza Suraya, Ekorini Listiowati, Deni W. Kurniawan, Abdillah Ahsan

**Affiliations:** 1 Department of Public Health, Universitas Muhammadiyah Prof. Dr. Hamka, Jakarta, Indonesia; 2 Department of Public Health, Universitas Islam Negeri Syarif Hidayatullah Jakarta, Jakarta, Indonesia; 3 Department of Family Medicine and Public Health, Universitas Muhammadiyah Yogyakarta, Yogyakarta, Indonesia; 4 Department of Economics, Universitas Indonesia, Jawa Barat, Indonesia

**Keywords:** cigarette smoking, COVID-19, secondhand smoke, developing countries

## Abstract

**INTRODUCTION:**

Indonesia is ranked fourth among countries with the highest smoking rates and has the highest number of male smokers globally. This study aimed to assess the association between cigarette smoking patterns and the severity of COVID-19 among patients in 15 Indonesian hospitals.

**METHODS:**

A cross-sectional study was conducted from April to August 2020 using medical records of 490 COVID-19 patients, including the history of their smoking patterns from 15 private referral hospitals in 5 provinces. The severity was defined based on the Guidelines on the Prevention and Control of COVID-19 issued by the Indonesian Ministry of Health, which was indicated by the care provided to patients, namely outpatient, inpatient, and Intensive Care Unit (ICU) services for mild, moderate, and severe symptoms. Smoking patterns were grouped based on adult tobacco use classifications of the Centers for Disease Control and Prevention (CDC). Univariate and bivariate analyses were performed.

**RESULTS:**

The results showed that 69.8% of respondents had not smoked cigarettes, 17.1% were active smokers, and 13.1% were former smokers. A significant difference was seen in the number of cigarettes smoked by patients in the ICU, inpatients, and outpatients, among current smokers and passive smokers (p=0.018 and p=0.005, respectively). Furthermore, there was no significant difference in the severity of COVID-19 among current smokers, former smokers, and non-smokers. The time from when smoking was stopped among former smokers was not associated with the severity of COVID-19.

**CONCLUSIONS:**

There was no significant difference in COVID-19 severity between groups of smokers. Passive smoking and the number of cigarettes smoked by smokers daily were associated with the severity of COVID-19. Smoke-free policies should be implemented continuously to protect people from the dangers of secondhand smoke.

## INTRODUCTION

Indonesia is among the countries severely affected by the COVID-19 pandemic. During 2020, data show that Indonesia recorded the most COVID-related deaths in South-East Asia^1^. Moreover, more than half of the cumulative number of confirmed cases was in Java Island. Therefore, with the highest number of new cases and deaths during mid 2021, Indonesia was considered Asia’s new COVID-19 epicenter^2^.

Smokers have increased airway expression of ACE-2, the entry receptor for the virus, and could have an increased risk of severe COVID-19 by activating a ‘cytokine storm’^3-5^. A previous study showed that smokers were 1.4 times more likely to have severe symptoms and approximately 2.4 times to be admitted to an Intensive Care Unit (ICU), need mechanical ventilation, or die, than non-smokers^6^. A similar study showed that patients who currently smoked had a five times greater risk of developing Acute Respiratory Distress Syndrome (ARDS) compared to non-smokers^7^.

Indonesia has the fourth highest smoking rate globally, with one-third (33.8%) active adult smokers by 2018 and the highest number of male smokers worldwide^8^. Several studies regarding the relationship between smoking and the severity of COVID-19 have been conducted. These studies did not examine smoking patterns in detail, such as frequency, duration of smoking, the number of cigarettes, and exposure to smoke, with the severity of COVID-19. Meanwhile, secondhand smoke exposure as a risk factor for severity or outcomes remained understudied^9^. This study examined the association between cigarette smoking patterns and the severity of COVID-19 among patients from 15 referral hospitals in Indonesia.

## METHODS

This study was conducted in 15 Muhammadiyah-’Aisyiyah-affiliated COVID-19 referral hospitals allocated in five provinces, namely DKI Jakarta, East Java, Central Java, Yogyakarta, and Central Kalimantan. The respondents were registered hospital patients who tested positive for COVID-19 using the reverse transcriptase polymerase chain reaction (PCR) tests during the laboratory examination from April to August 2020. In total, 490 respondents were recruited.

The outcome was defined based on the fifth edition of the Guidelines on the Prevention and Control of COVID-19 issued by the Ministry of Health of Indonesia. The guidelines state that patients with the virus without symptoms could undergo isolation at home. The patients suffering from moderate symptoms needed to be hospitalised, while those with severe symptoms required treatment in an ICU. Cigarette smoking status was grouped according to the CDC adult tobacco use classification^10^. Information on respondents’ demographic data, clinical characteristics, and the given treatment, were retrieved from the hospital records, whereas nurses collected data on smoking habits after patient discharge at each hospital. Univariate and bivariate analyses were conducted to analyse the association between smoking behaviour with the severity of COVID-19 using statistical analysis software.

## RESULTS

Among the 490 respondents, the female participants (n=260; 53.1%) were more than the male (n=230; 46.9%), and 83.2% received inpatient services. However, 69.8%, 17.1%, and 13.1% were non-smokers, active smokers and former smokers. Regarding exposure to cigarette smoke, 60.4% of respondents were occasionally exposed, while 17.6% had not been exposed to cigarette smoke. The average smoker consumed 10 cigarettes daily, or around 4 pack-years.

Our study observed that there were differences in the severity of COVID-19 among different age groups, with those aged ≥60 years having a higher proportion of ICU treatment compared to the younger age group (<60 years), 9.7% vs 3.1%, respectively. The results showed no significant relationship between respondents’ smoking status and the severity of COVID-19, (p=0.133). The time since quitting smoking among former smokers showed no significant relationship with COVID-19 severity (p=0.657). However, a significant difference was observed in the number of daily cigarettes smoked by patients in the ICU (14.8 ± 4.3), inpatient care (10.0 ± 4.4), and outpatient care (7.7 ± 6.0) (p=0.023). Pack-years cigarette smoking was also associated with the severity of COVID-19 (p=0.009). COVID-19 severity was also higher among those who reported to be exposed to passive smoking (p=0.005), as noted in Table 1.

**Table 1:** Factors associated with the severity of COVID-19 among participants from 15 private hospitals in Indonesia, 2020 (N=490)

*Variable*	*Total*		*The severity of COVID-19 based on the care received by the patient*						*p*
			*Intensive Care Unit*		*Inpatient*		*Outpatient*		
	*n*	*%*	*n*	*%*	*n*	*%*	*n*	*%*	
**Total**	490	100.0	22	6.4	416	83.2	52	10.4	
**Sex**									0.618
Male	230	46.9	12	5.2	196	85.2	22	9.6	
Female	260	53.1	10	3.8	220	84.6	30	11.5	
**Age** (years)									0.003
≥60	103	21.0	10	9.7	85	82.5	8	7.8	
<60	387	79.0	12	3.1	331	85.5	44	11.4	
**Smoking status**									0.133
Current	84	17.1	5	6.0	68	81.0	11	13.1	
Former	64	13.1	5	7.8	57	89.1	2	3.1	
Never	342	69.8	12	3.5	291	85.1	39	11.4	
**Years from quitting smoking** (former smokers)									0.657
>5	28	5.7	1	3.6	26	92.9	1	3.6	
1–5	24 4.9	2 8.3	21	87.5	1	4.2	1	4.2	
<1	12	2.4	2	16.7	10	83.3	0	0.0	
**Smoking duration** (years), Mean ± SD	29.7 ± 13.5		31.8 ± 21.9		29.8 ± 13.2		28.2 ± 12.0		0.888
**Daily number of cigarettes smoked,** Mean ± SD	10.0 ± 4.8		14.8 ± 4.3		10.0 ± 4.4		7.7 ± 6.0		0.023
**Pack-years cigarette smoking,** Mean ± SD	4.1 ± 8.9		9.3 ± 15.3		4.0 ± 8.6		2.5 ± 6.8		0.009
**Exposure to cigarette smoke**									0.005
Often	108	22.0	11	10.2	82	75.9	15	13.9	
Sometimes	296	60.4	9	3.0	262	88.5	25	8.4	
Seldom/never	86	17.6	2	2.3	72	83.7	12	14.0	

Duration of smoking, number of daily cigarettes smoked, and pack per year cigarettes smoking were tested by ANOVA.

## DISCUSSION

Our research is in line with previous studies reporting that elderly patients with COVID-19 were more likely to progress to severe disease^11^. The current smoking rate of COVID-19 patients was lower than the overall smoking rate of the population in Indonesia. This could be because there were more females in the sample. However, based on Indonesian basic health research in 2018, the prevalence of female smokers was much lower than that of male smokers (1.9% vs 55.8%, respectively)^8^.

Although this study found that the proportion of smokers who received ICU treatment was greater than those who did not, the significance test obtained no significant relationship between smoking status and COVID-19 severity. Previous studies also reported no differences in COVID-19 outcomes between the groups^12^. However, differences were found in the severity between groups based on exposure to cigarette smoke. Secondhand smoke exposure is a known risk factor for cardiovascular diseases and chronic lung disease^13^. A previous study showed that exposure to secondhand smoke may also be associated with an increased risk for infection^11^.

Furthermore, we identified a significant relationship between the average number of cigarettes consumed and the severity of COVID-19. Moreover, on average, patients in the ICU smoked more cigarettes per day than those who received inpatient and outpatient care. A similar previous study showed that heavy smokers had a 7.0 times more risk of having COVID-related death compared to non-smokers^14^. A previous study also indicated that smoking intensity was associated with ICU admission^15^.

### Limitations

Despite these findings it is important to note that the study’s locations were private hospitals, and not all patients were represented. This is a cross-sectional study, and the result cannot be presented within a causal framework. Critically ill COVID-19 patients who had already died before being admitted to hospital were not sampled. Since our results did not control for participant age, results may differ in age adjusted analyses. However, it is significant to study the impact of secondhand smoke and smoking habits among patients in multi-center hospital settings, with a longitudinal study design.

## CONCLUSIONS

The study showed that secondhand smoke and the number of cigarettes smoked daily were related to the severity of COVID-19. The protection of people from smoke exposure through smoke-free policy programs needs to be continued and strengthened, especially during respiratory pandemics.

## Data Availability

The data supporting this research are available from the authors on reasonable request.
